# Real-World, Observational, Retrospective Study to Evaluate the Effectiveness and Safety of Treatment with Sorafenib in Patients with Advanced Hepatocellular Carcinoma

**DOI:** 10.3390/curroncol31110500

**Published:** 2024-11-01

**Authors:** Angélica Richart Csipak, Leonardo G. da Fonseca, Rossana Verónica Mendoza López, Maria Del Pilar Estevez-Diz

**Affiliations:** 1 Oncology, Instituto do Cancer do Estado de Sao Paulo, Faculdade de Medicina da Universidade de Sao Paulo, São Paulo 01246-000, Brazil; l.fonseca@fm.usp.br (L.G.d.F.); maria.pilar@hc.fm.usp.br (M.D.P.E.-D.); 2 Comprehensive Center for Precision Oncology C2PO, Center for Translational Research in Oncology, Instituto do Cancer do Estado de Sao Paulo, Faculdade de Medicina da Universidade de Sao Paulo, São Paulo 01246-000, Brazil; rossana.veronica@hc.fm.usp.br

**Keywords:** advanced hepatocellular carcinoma, sorafenib, real-world, survival, effectiveness

## Abstract

Background: Hepatocellular carcinoma (HCC) accounts for approximately 90% of liver cancer cases. Sorafenib, the first drug to demonstrate survival benefits for advanced HCC, was validated through the SHARP randomized clinical trial (RCT). While RCTs are essential for assessing new therapies, real-world studies provide additional insights into their effectiveness in routine clinical practice. This study aimed to evaluate sorafenib’s real-world effectiveness by analyzing overall survival (OS) and the time to radiological and symptomatic progression. Methods: Data from 368 patients treated with sorafenib at a Brazilian Cancer Center between 2009 and 2020 were retrospectively reviewed. Results: The median OS was 9.6 months, and the time to radiological progression was 5.3 months, similar to the SHARP trial. However, the time to symptomatic progression was shorter (2.3 months) than the SHARP study (4.1 months). In terms of safety, 27.4% of patients presented clinically relevant toxicities, and 24.5% needed to discontinue treatment due to toxicity. Conclusions: Overall, sorafenib demonstrated effectiveness in the studied population, with OS and radiological progression times comparable to SHARP study results. The difference in symptomatic progression may be due to the study’s retrospective nature and limitations.

## 1. Introduction

In 2020, liver cancer accounted for 4.7% (905,677 cases) of new cancer cases, making it the sixth most common malignancy, and the third leading cause of cancer-related deaths (8.3%) worldwide [1]. In Brazil, the estimated number of new cases in 2020 was 12,674 [2]. There is a gender unbalance in the incidence of liver cancer globally, with rates 2–3 fold higher among men than women [3,4]. In Brazil, the estimated incidence in 2020 was also higher in males, with a proportion of about 1.5:1 (men:women) [2].

The most common type of primary liver cancer is hepatocellular carcinoma (HCC), which represents approximately 90% of cases [5]. The main risk factors associated with the development of HCC include chronic infection by hepatitis B (HBV) and hepatitis C (HCV) viruses, alcohol abuse, and metabolic-associated steatotic liver disease [4,6].

For cases diagnosed at an advanced stage or with progression after locoregional therapy, systemic treatment is indicated, aiming at slowing tumor progression, increasing survival, and controlling symptoms [5,7,8].

The first agent to show benefit in patients with advanced HCC was sorafenib, according to data published in 2008. Until then, no systemic therapy had demonstrated a positive impact on advanced HCC [9]. For about a decade, sorafenib remained the only approved first-line treatment and the standard of care for advanced hepatocellular carcinoma [5]. However, the treatment landscape has recently evolved with the approval of additional first and second-line systemic treatments [10]. In addition to sorafenib, atezolizumab (in combination with bevacizumab), durvalumab (in combination with tremelimumab), lenvatinib, cabozantinib, ramucirumab, regorafenib, and nivolumab are currently approved by the Brazilian regulatory agency for HCC, with the last four indicated only for patients previously treated with sorafenib [11,12,13,14,15,16,17]. 

Sorafenib is an oral drug that inhibits multiple intracellular kinases (c-CRAF, BRAF, and mutated BRAF) and cell surface receptors (KIT, FLT-3, RET, RET/PTC, VEGFR-1, VEGFR-2, VEGFR-3, and PDGFR-beta), which are involved in the activation of intracellular signaling pathways, leading to the growth and proliferation of tumor cells, in angiogenesis and in the inhibition of apoptosis [7,9,18,19,20]. 

Sorafenib was shown to provide survival benefit in a randomized, phase III, prospective, placebo-controlled study that evaluated 602 patients with preserved liver function (Child–Pugh A) and preserved functionality (Eastern Cooperative Oncology Group - ECOG 0–2) diagnosed with unresectable HCC [9]. 

Although randomized clinical trials are considered the gold standard for evaluating the safety and efficacy of new therapeutic agents, their strict eligibility criteria and the standards used to eliminate possible biases, may lead to the inclusion of a population that is not representative of patients found in clinical practice. In this context, real-world studies are important tools that provide relevant information to complement and expand upon data previously obtained in randomized clinical studies [21]. According to the Food and Drug Administration (FDA), “*real-world evidence is the clinical evidence about the usage and potential benefits or risks of a medical product derived from analysis of real-world data*” [22]. In recent years, real-world studies have been conducted around the world to evaluate and validate data on the efficacy/effectiveness and safety of sorafenib in patients with advanced HCC, with heterogeneous findings depending on the assessed context. In addition, the benefit of maintaining this treatment after radiological progression is questioned in the absence of symptomatic deterioration, as predicted in the initial studies. In the Brazilian context, assessments regarding the radiological and, especially, symptomatic time to progression (TTP) are scarce.

In this sense, we proposed a real-world, observational, retrospective study to assess the effectiveness and safety of treatment with sorafenib in patients with advanced HCC.

Thus, the general objective of the study is to evaluate the effectiveness and safety, in the real-world context, of the use of sorafenib in the advanced HCC in patients treated at Instituto do Cancer do Estado de Sao Paulo (ICESP). In this study, the objectives are to evaluate time to progression (TTP); to evaluate comparatively the overall survival (OS) of patients treated until the occurrence of symptomatic progression versus those treated until the radiological progression; to assess the OS according to ECOG performance status (ECOG-PS), Barcelona Clinic Liver Cancer System (BCLC) classification, Child–Pugh classification, all at the beginning of treatment with sorafenib and etiology (HCV, HBV, alcoholism, nonalcoholic steatohepatitis - NASH, cryptogenic cirrhosis); to compare the OS of the total sample with the results available in the SHARP study; to assess the main reason for discontinuing treatment (radiological progression, symptomatic progression, or safety) and safety.

## 2. Patients and Methods

This is a real-world, observational, retrospective study to evaluate the effectiveness and safety of treatment with sorafenib in patients with advanced HCC. Retrospective data were collected from patients of both sexes, aged 18 years or older at the time of starting treatment with sorafenib, diagnosed with advanced HCC, whose prescribed treatment was sorafenib, with treatment starting between 2009 (the year in which the use of sorafenib began in the institution) and 2020. The pre-defined exclusion criteria were as follows: participants diagnosed with other invasive neoplasms in the five years before the diagnosis of HCC, patients who started sorafenib treatment at other institutions and were referred to ICESP for treatment continuation, and patients who received experimental drugs for the treatment of HCC and/or its complications. 

During the period evaluated by this study, sorafenib was the first-line treatment available for candidates for systemic therapy at the institution. Sorafenib was administered orally at an initial dose of 400 mg twice daily, which could be adjusted based on the type and severity of adverse events. Clinical and laboratory assessments were typically performed at the beginning of treatment and monthly, while radiological evaluations were generally conducted bi-monthly. Treatment typically continued until symptomatic progression, radiological progression, treatment intolerance, or death. Information was obtained through a database of electronic medical records in Tasy (Philips Tasy—Health Management Solution) at ICESP. The data were obtained after approval of the project by the Ethics Committee of HCFMUSP by opinion number 4,681,412 of 29 April 2021. Data collection started in 2021. 

OS was defined as the time between the start of treatment with sorafenib and the occurrence of death from any cause, according to the medical records.

TTP was defined as the time between the start of treatment with sorafenib and disease progression based on the assessment documented by the treating physician in each participant’s medical record. This assessment may have considered imaging tests, signs, and symptoms; however, for this project, only the final evaluation of the physician was used after they analyzed all available clinical information regarding the patient. TTP was evaluated using two different parameters: time to radiological progression and time to symptomatic/clinical progression. The safety assessment regarding treatment with sorafenib was evaluated according to two variables: the percentage of participants who needed to interrupt treatment due to toxicity and the proportion of participants who showed good tolerability to treatment with sorafenib, according to medical evaluation available in the medical record.

Clinical and epidemiological characteristics were analyzed using descriptive statistics. Continuous variables were expressed as means, medians, standard deviation, and minimum and maximum values; categorical variables were expressed as absolute and relative frequencies. Survival curves were estimated using the Kaplan–Meier method and compared using the log-rank test. The Cox regression method was used to estimate the hazard ratio (HR) and 95% confidence intervals and, thus, evaluate the interaction between potential prognostic factors and survival. The significance level adopted was 5% for all hypothesis tests. The analyses were conducted using SPSS for Windows v.25 software.

## 3. Results

### 3.1. Patient Characteristics

The initial survey identified 494 patients who had been prescribed sorafenib between 2009 and 2020. Of these, 126 were not included in the analysis, 78 had diagnoses other than HCC, 44 had not started treatment with sorafenib, 2 withdrew for personal reasons, 1 started treatment at another institution, and 1 was under 18 years old at the time of treatment initiation. 

**Figure 1:**
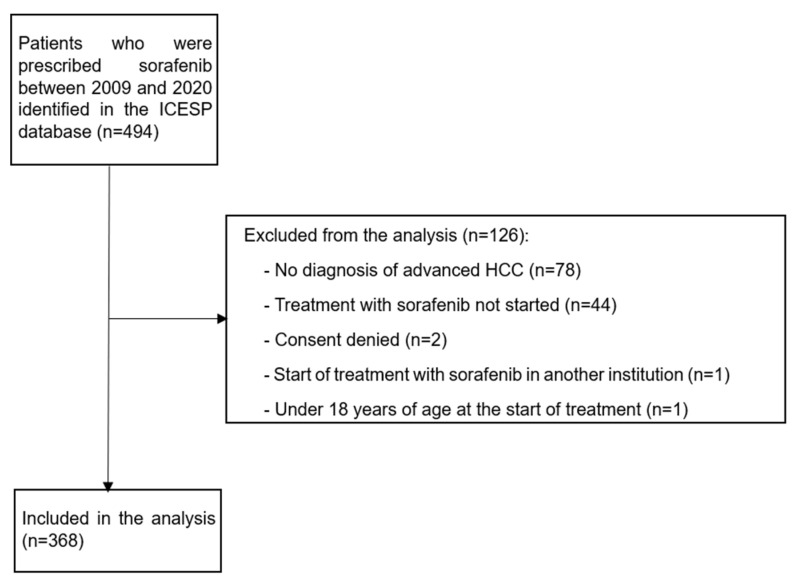
Flowchart: screening of patients. ICESP: Instituto do Cancer do Estado de Sao Paulo; HCC: hepatocellular carcinoma.

Thus, 368 participants were eligible for the study (Figure 1). At the time of data collection, 326 (88.58%) of the study participants had already deceased.

In total, 279 (75.8%) were male and 89 (24.2%) were female, resulting in a male-to-female ratio of 3:1. The mean age at the start of sorafenib treatment was 61.5 years. Regarding etiology, most participants (51.1%) had HCV infection. At the time of starting treatment with sorafenib, most participants (57.6%) had an ECOG-PS of 0, (83.1%) had a preserved liver function (Child–Pugh A), and were classified as BCLC stage C (75.3%) (Table 1).

### 3.2. Duration of Treatment

The mean duration of treatment with sorafenib in the analyzed sample was 7 months (220.8 days), with a median of 4 months (123 days). A total of 351 participants (95.4% of the analyzed sample) had discontinued treatment at the time of data collection. Of these, 70.9% discontinued treatment due to disease progression, 24.5% due to toxicity, 4% due to being lost to follow-up, and 0.2% for other reasons.

### 3.3. Progression Data

Among the participants who showed progression during treatment with sorafenib, the majority had radiological progression detected by the physician monitoring the patient through imaging exams (217, or 75.1%). Only 72 patients (24.9%) showed symptomatic/clinical progression in the absence of radiological progression, according to the medical records.

Patients who presented radiological progression had a longer time to progression compared to patients who presented symptomatic/clinical progression (Table 2).

### 3.4. Overall Survival

Table 3 presents the results related to the OS of the total study population and according to the type of progression, performance status (ECOG), BCLC stage, Child–Pugh classification, and etiology.

**Figure 2:**
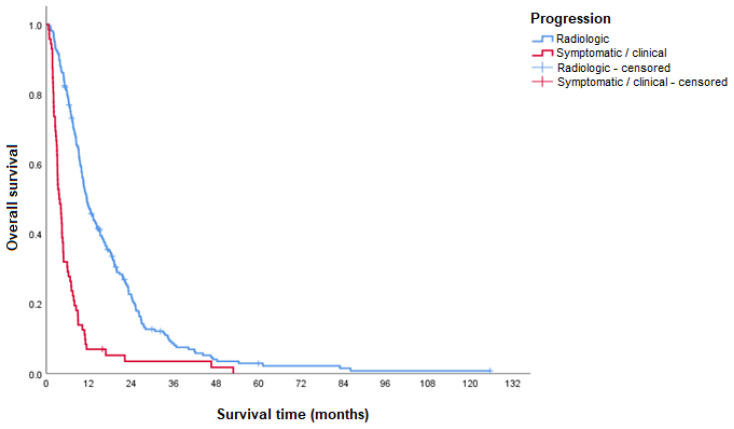
OS according to the type of progression at the time of treatment discontinuation.

It was observed that there was better survival in the subgroup of participants with radiological progression compared to those with symptomatic/clinical progression at the time of discontinuation of sorafenib treatment (Table 3 and Figure 2).

**Figure 3:**
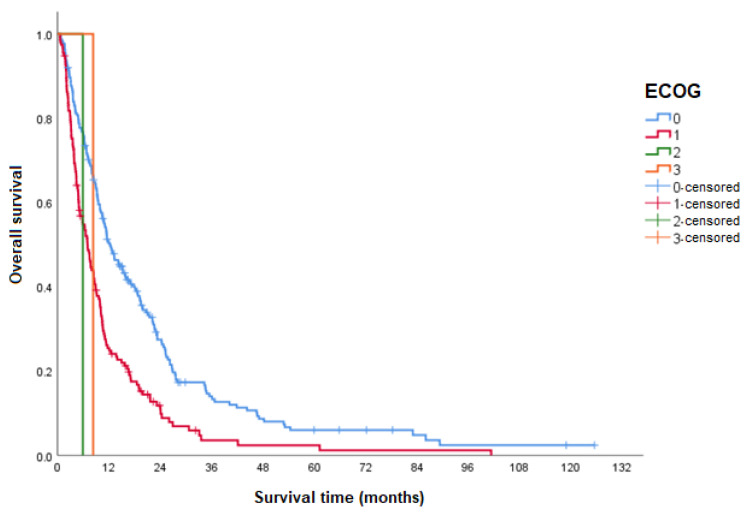
OS according to the ECOG performance status. ECOG: Eastern Cooperative Oncology Group.

Patients who had been classified as ECOG-PS 0 had a higher median OS compared to patients classified as ECOG-PS 1 at the beginning of treatment (Table 3 and Figure 3).

**Figure 4:**
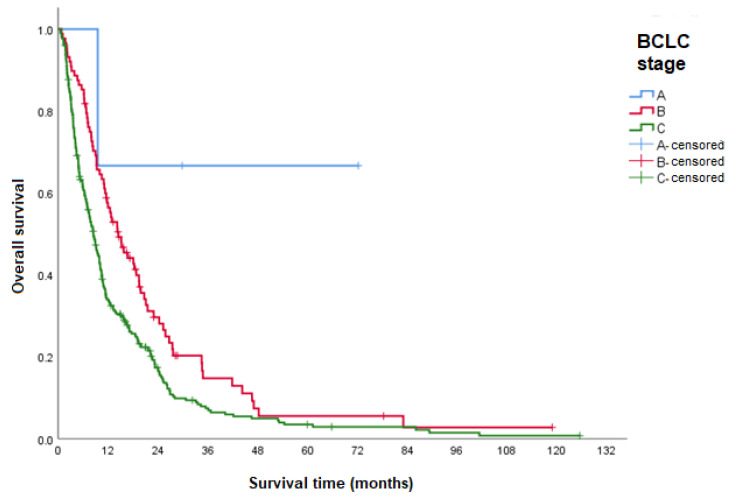
OS according to BCLC stage. BCLC: Barcelona Clinic Liver Cancer System.

OS was assessed according to the BCLC stage at the start of sorafenib treatment. Comparison was only possible between stages B and C due to the small number of patients classified in the other stages. A higher median OS was demonstrated for patients in stage B than for those classified as stage C (Table 3 and Figure 4).

**Figure 5:**
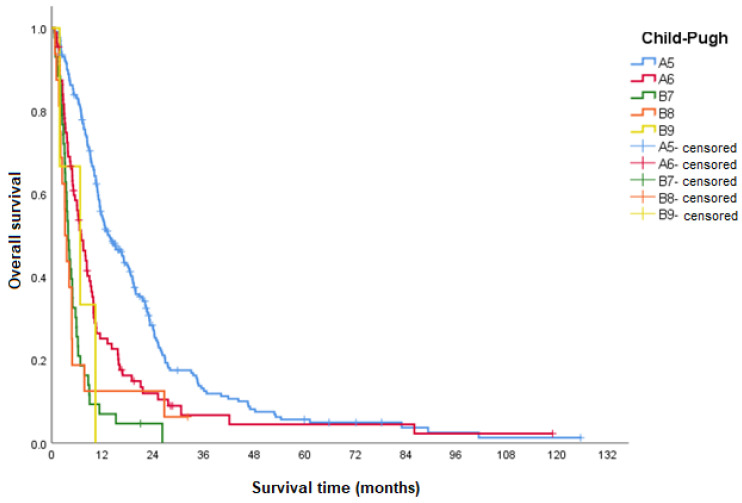
OS according to Child–Pugh class.

It was observed that the better the Child–Pugh classification, the higher the median OS, with a statistically significant difference between the groups. The only exception was related to the B(9) category; however, there was a low number of individuals in the sample who presented this category (*n* = 3), which did not allow for a reliable analysis of this subgroup (Table 3 and Figure 5).

**Figure 6:**
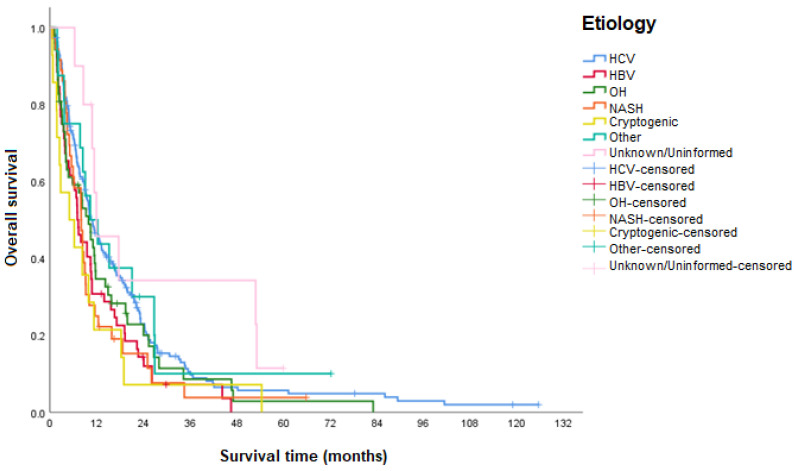
OS according to etiology. HCV: hepatitis C virus; HBV: hepatitis B virus; OH: alcohol; NASH: nonalcoholic steatohepatitis.

Patients with HCV had higher survival compared to the other etiologies evaluated. When compared to the HCV group, participants with HBV, NASH, and cryptogenic cirrhosis demonstrated a significantly worse median OS (Table 3 and Figure 6).

**Figure 7:**
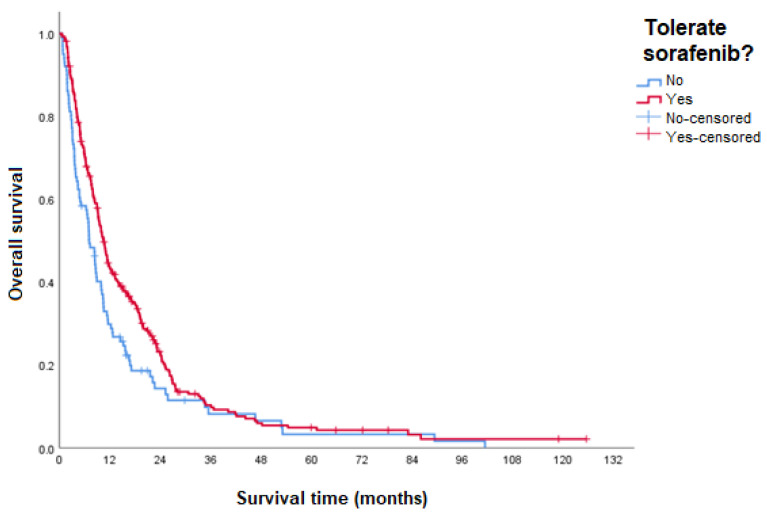
OS according to sorafenib tolerability.

Regarding safety, according to the information in the medical records, 267 participants tolerated treatment with sorafenib, while 101 did not demonstrate good tolerability (considering the use of at least 400 mg—2 tablets per day), according to the assessment in the medical records, taking into account the need for dose adjustment or occurrence of toxicity. Additionally, the influence of the good tolerability of the treatment on the participants’ OS was evaluated. Thus, participants who had good tolerability to sorafenib treatment had a higher median OS (10.5 months, 95% CI 9.2–11.8) compared to those who did not have good tolerability (7.1 months, 95% CI 5.5–8.7) (*p* = 0.011) (Figure 7).

## 4. Discussion

The effectiveness of sorafenib treatment was assessed using the variables time to progression and overall survival. The results were compared with those obtained in the SHARP trial, which led to the approval of sorafenib for the treatment of advanced HCC globally. Such comparisons were conducted to verify whether the results of this real-world study would reproduce the same findings obtained through a randomized clinical trial.

The time to radiological progression observed in the sorafenib-treated group in the SHARP randomized clinical trial was 5.5 months (95% CI 4.1–6.9), whereas in this real-world study, it was 5.3 months (95% CI 4.7–6.0) [9]. Thus, the results demonstrated great similarity, indicating reproducibility in the results of this variable. The time to symptomatic/clinical progression presented in the SHARP study was 4.1 months (95% CI 3.5–4.8), while in this study, it was 2.3 months (95% CI 1.3–3.4), demonstrating a significant difference between the results obtained for this analysis [9].

Additionally, similarity was demonstrated between the results obtained for the time to radiological progression, but a significant difference was noted in the comparison of the results found for time to symptomatic/clinical progression. Our study included patients with Child–Pugh B and real-world comorbidities, who are at higher risk for hepatic decompensation. This group of patients may impact the short time-to-symptomatic progression found. 

Regarding time to symptomatic/clinical progression, in addition to being a subjective analysis, it was not possible to implement in this study the same tool used in the SHARP study to direct such assessment. In the SHARP study, symptomatic progression was defined by varying scores when applying the standardized Functional Assessment of Cancer Therapy Hepatobiliary Symptom Index 8 (FHSI8) questionnaire or classification 4 on the ECOG scale. In the present study, due to its retrospective design, there was no possibility of applying a questionnaire for this purpose. For this reason, it would not be possible to consider this information to define the moment of clinical/symptomatic progression.

Given the limitations discussed, the result of time to symptomatic progression in this study was obtained in a quite unique way from obtaining this information in the SHARP study. Therefore, this is the reason for the discrepancy in the results of this variable between studies.

The median OS of the overall study population was evaluated in comparison to the sorafenib-treated sample in the SHARP randomized clinical trial. As shown in Table 3, the sample in this study had a median OS of 9.6 months (95% CI 8.5–10.7), while the group treated with sorafenib in the SHARP study had a median OS of 10.7 months (95% CI 9.4–13.3) [9]. Although the present study demonstrates a median OS of approximately 1 month less than that found in the randomized study, crossing their confidence intervals indicates an overlap in the estimates. Therefore, the difference found was not considered statistically significant. Possibly, the difference found between the population of this study and the group treated with sorafenib in the SHARP study is due to the different methodologies between a real-world study and a randomized clinical trial.

RCTs are considered the gold standard for proving the efficacy of a new molecule precisely due to all the controls that aim to reduce biases that could influence the efficacy analysis, such as strict eligibility and discontinuation criteria, and control of treatment adherence. Therefore, randomized clinical trials have high internal validity. On the other hand, these studies may have low external generalizability, since in the real context of clinical practice it is not feasible to use so many controls when choosing treatment and monitoring the patient.

In real-world studies, it is possible to verify the effectiveness of a treatment exactly in the real context of clinical practice. This includes a heterogeneous sample of patients, with different concomitant pathologies and possible failures in adherence to treatment. For this reason, the fact that OS in this study was lower than that of the group treated with sorafenib in the SHARP study was expected.

In the real-world context, some study publications also evaluated the effectiveness of sorafenib in HCC around the world. However, there is no consistency between the effectiveness results. OS analysis, for example, varies from approximately 5 to 32 months across these studies. One factor that causes this variation in results is the difference in baseline characteristics, context, and clinical practice in each study/institution. Among real-world studies, clinical practice also varies regarding medication maintenance even in patients who demonstrate treatment intolerance/toxicity [23,24,25,26,27].

The median OS of patients with radiological progression at the discontinuation of sorafenib treatment was significantly higher than the OS of those with symptomatic progression (HR 3.1). Clinical deterioration in HCC is usually associated with liver dysfunction and cirrhosis complications, highlighting the poor prognosis associated with symptomatic progression. 

OS was also assessed according to the ECOG performance scale at the beginning of sorafenib treatment. Thus, it was observed in this study that patients classified as ECOG 0 at the beginning of treatment had a significantly higher median OS than those who had been classified as ECOG 1 (HR 1.8). Although, as previously discussed, the information collected on the ECOG scale may not have been completely reliable in reality; the result obtained in this analysis is consistent with what was expected, since patients with better functional capacity at the beginning of treatment tended to have a longer median OS time.

Patients with BCLC stage B (intermediate) at the beginning of sorafenib treatment had a significantly higher median OS than those with BCLC stage C (advanced) (HR 1.5, 95% CI 1.2–2.0). The result obtained in this analysis is in line with the literature [25,26,27] and as expected, since patients in more advanced stages had a shorter median OS.

As expected, patients with better scores on the Child–Pugh classification of liver disease severity had higher median OS. Thus, patients classified as Child–Pugh A(5), A(6), B(7), B(8), and B(9) had, respectively, an OS in months of 13.7 (HR 1); 7.0 (HR 1.8); 3.9 (HR 4.0); 3.2 (HR 3.2); and 6.7 (HR 3.3). The only exception regarding the decrease in median OS according to the highest scores was about category B(9), probably due to the low number of individuals in the sample who presented this category (*n* = 3), which did not allow for a reliable analysis of this subgroup.

Regarding the etiology, the subgroup diagnosed with HCV had the highest observed median OS, followed by alcoholism, NASH, HBV, and cryptogenic cirrhosis. No rationale explains this difference between the etiologies in OS, with the possibility that the differences between the subgroups may have been found by chance.

The median duration of treatment with sorafenib in the population analyzed was approximately 4 months, and the main reason for the discontinuation of treatment was disease progression (70.9%), followed by treatment toxicity (24.5%). In the randomized SHARP study, the median duration of treatment was around 5 months, and the main reason for discontinuation was the occurrence of adverse events, both in the group treated with sorafenib (38.05%) and in the group that received placebo (37.19%) [9]. The highest proportion of treatment interruption due to the occurrence of adverse events/toxicity is expected in randomized clinical trials, since the search for adverse events is commonly carried out actively in this type of study through targeted and frequent questions, thus increasing the chance of capturing this information and detecting the need to interrupt treatment due to possible toxicity. Additionally, as the high discontinuation due to adverse events occurred in both groups (active and placebo) in the randomized clinical study, there is the possibility that symptoms related to disease progression were considered adverse events, leading to treatment interruption, possibly due to strict discontinuation criteria common in phase 3 clinical trials.

Additionally, it was observed that 27.4% of the patients evaluated did not show good tolerability to treatment with sorafenib, although not all of them required interruption of treatment for this reason.

The impact of safety on the survival of patients in the sample was evaluated. Thus, participants who had good tolerability to sorafenib treatment had a significantly higher median OS than those who did not. Such a response was expected, since patients who do not have good tolerability may require dose reduction, a temporary discontinuation of treatment until recovery from adverse events, or a permanent interruption of treatment. Thus, it is possible to infer that the occurrence of adverse events can directly and negatively impact the effectiveness of the treatment.

In recent years, there has been a growing awareness and acceptance of real-world studies by different stakeholders, including doctors, pharmaceutical companies, regulators, and patients. Several regulatory authorities have developed guides and guidelines for the use of real-world studies and data while recognizing several challenges in collecting and analyzing real-world data [28].

A key challenge in conducting retrospective real-world studies is overcoming limitations related to data standardization, availability, and granularity. In this study, these limitations posed challenges to the analysis of both symptomatic progression and safety outcomes. A challenge of this study regarding the assessment of safety and tolerability is the retrospective design, which made it difficult to precisely determine which adverse events led to treatment discontinuation or identified toxicity. In many cases, the medical records lacked detailed descriptions of the specific adverse events that were critical in determining the participant’s inability to tolerate the treatment.

In the present study, it was possible to observe the importance of real-world data to complement the information obtained through randomized clinical studies. In the case in question, it was possible to verify a decrease, although not significant, in the survival of patients treated with sorafenib, in addition to an alignment between the times until radiological progression.

## 5. Conclusions

The results of the effectiveness analysis indicate that treatment with sorafenib was effective in the population evaluated. Based on the results of this study, patients who seem to benefit the most from sorafenib treatment, in terms of OS, are those who had an ECOG score of 0 at the start of treatment, were classified as Child–Pugh A, and whose etiology was HCV or alcoholism. 

OS and time to radiological progression showed no significant difference from the results of the SHARP study, demonstrating an alignment between real-world results and those from a randomized clinical trial in the case of sorafenib treatment. The time to symptomatic/clinical progression showed a significant difference in the SHARP study, but this result must be evaluated with caution, considering the limitations of retrospective analysis. 

All factors analyzed (type of progression, ECOG, BCLC, Child–Pugh, and etiology) demonstrated an influence on the sample’s median OS. Regarding safety, a significant portion of the sample did not tolerate treatment with sorafenib well, requiring early discontinuation of treatment, which negatively influenced OS. 

In the present study, it was possible to observe the importance of real-world data to complement the information obtained through randomized clinical studies.

## Figures and Tables

**Table 1:** Baseline characteristics of participants.

Characteristic (*n* = 368)	*n*	%
**Sex**		
Male	279	75.8
Female	89	24.2
**Age (years)**		
Mean (SD)	61.5 (10.4)	
Median (minimum-maximum)	62.3 (19–86.3)	
**Etiology**		
HCV	188	51.1
HBV	52	14.1
Alcohol only	52	14.1
NASH	36	9.8
Cryptogenic cirrhosis	14	3.8
Other *	16	4.3
Unknown/Uninformed	10	2.7
**ECOG**		
0	212	57.6
1	154	41.8
2	1	0.3
3	1	0.3
**Child–Pugh class**		
A (5)	218	59.2
A (6)	88	23.9
B (7)	43	11.7
B (8)	16	4.3
B (9)	3	0.8
**BCLC stage**		
A	3	0.8
B	88	23.9
C	277	75.3

SD: standard deviation; HCV: hepatitis C virus; HBV: hepatitis B virus; NASH: nonalcoholic steatohepatitis; ECOG: Eastern Cooperative Oncology Group; BCLC: Barcelona Clinic Liver Cancer System; * hemochromatosis (*n* = 8), schistosomiasis (*n* = 6), alpha-1 antitrypsin deficiency (*n* = 1), and autoimmune disease (*n* = 1).

**Table 2:** Time to radiological and symptomatic/clinical progression.

Time to Progression (TTP)	Median (95% CI) (Months)
Time to radiological progression	5.3 (4.7–6.0)
Time to symptomatic/clinical progression	2.3 (1.3–3.4)

TTP: time to progression; 95% CI: 95% confidence interval.

**Table 3:** Overall survival.

Overall Survival (OS)	Median (95% CI) (Months)	*p* Value	HR (95% CI)
**General study population**			
**OS**	9.6 (8.5–10.7)		
**OS according to the type of progression at the time of treatment discontinuation**			
Radiological progression	11.5 (9.7–13.2)	<0.001	1
Symptomatic/clinical progression	3.6 (2.6–4.7)		3.1 (2.3–4.1)
**OS according to the ECOG performance status**			
ECOG 0	12.3 (9.9–14.6)	<0.001	1
ECOG 1	7.0 (5.6–8.4)		1.8 (1.4–2.3)
ECOG 2	5.9		3.4 (0.5–24.1)
ECOG 3	8.3		2.2 (0.3–16.3)
**OS according to BCLC stage**			
BCLC B (intermediate)	14.5 (10.5–18.5)	<0.001	1
BCLC C (advanced)	8.5 (7.2–9.8)		1.5 (1.2–2.0)
**OS according to Child–Pugh class**			
Child–Pugh A(5)	13.7 (10.4–17.0)	<0.001	1
Child–Pugh A(6)	7.0 (5.3–8.8)	<0.001	1.8 (1.4–2.4)
Child–Pugh B(7)	3.9 (3.2–4.7)	<0.001	4.0 (2.8–5.7)
Child–Pugh B(8)	3.2 (2.3–4.0)	<0.001	3.1 (1.8–5.3)
Child–Pugh B(9)	6.7 (0–14.5)	0.037	3.3 (1.1–10.7)
**OS according to etiology**			
HCV	10.5 (8.4–12.7)	0.023	1
HBV	7.1 (5.7–8.4)	0.019	1.5 (1.1–2.0)
Alcohol only	9.9 (6.9–12.9)	0.202	1.2 (0.9–1.7)
NASH	8.0 (7.3–8.7)	0.049	1.5 (1.0–2.1)
Cryptogenic cirrhosis	5.0 (0.0–11.4)	0.040	1.8 (1.0–3.1)
Other	10.3 (4.1–16.6)	0.719	0.9 (0.5–1.6)
Unknown/Uninformed	12.0 (3.0–20.9)	0.171	0.6 (0.3–1.2)

OS: overall survival; HR: hazard ratio; 95% CI: 95% confidence interval; ECOG: Eastern Cooperative Oncology Group; BCLC: Barcelona Clinic Liver Cancer System; HCV: hepatitis C virus; HBV: hepatitis B virus; NASH: nonalcoholic steatohepatitis.

## Data Availability

The raw data are unavailable due to privacy and ethical restrictions.
